# Hydrocephalus: historical analysis and considerations for treatment

**DOI:** 10.1186/s40001-022-00798-6

**Published:** 2022-09-01

**Authors:** Alexandra Hochstetler, Jeffrey Raskin, Bonnie L. Blazer-Yost

**Affiliations:** 1 Department of Biology, Indiana University Purdue University Indianapolis, Indianapolis, IN USA; 2 Division of Pediatric Neurosurgery, Ann and Robert H. Lurie Children’s Hospital, Chicago, IL USA; 3 Department of Neurosurgery, Northwestern University Feinberg School of Medicine, Chicago, IL USA

**Keywords:** Hydrocephalus, Cerebrospinal fluid, Drug development, Pathophysiology, Preclinical research, Mechanisms

## Abstract

Hydrocephalus is a serious condition that affects patients of all ages, resulting from a multitude of causes. While the etiologies of hydrocephalus are numerous, many of the acute and chronic symptoms of the condition are shared. These symptoms include disorientation and pain (headaches), cognitive and developmental changes, vision and sleep disturbances, and gait abnormalities. This collective group of symptoms combined with the effectiveness of CSF diversion as a surgical intervention for many types of the condition suggest that the various etiologies may share common cellular and molecular dysfunctions. The incidence rate of pediatric hydrocephalus is approximately 0.1–0.6% of live births, making it as common as Down syndrome in infants. Diagnosis and treatment of various forms of adult hydrocephalus remain understudied and underreported. Surgical interventions to treat hydrocephalus, though lifesaving, have a high incidence of failure. Previously tested pharmacotherapies for the treatment of hydrocephalus have resulted in net zero or negative outcomes for patients potentially due to the lack of understanding of the cellular and molecular mechanisms that contribute to the development of hydrocephalus. Very few well-validated drug targets have been proposed for therapy; most of these have been within the last 5 years. Within the last 50 years, there have been only incremental improvements in surgical treatments for hydrocephalus, and there has been little progress made towards prevention or cure. This demonstrates the need to develop nonsurgical interventions for the treatment of hydrocephalus regardless of etiology. The development of new treatment paradigms relies heavily on investment in researching the common molecular mechanisms that contribute to all of the forms of hydrocephalus, and requires the concerted support of patient advocacy organizations, government- and private-funded research, biotechnology and pharmaceutical companies, the medical device industry, and the vast network of healthcare professionals.

**Supplementary Information:**

The online version contains supplementary material available at 10.1186/s40001-022-00798-6.

## Introduction

The first known reports of “water” around the brain of macrocephalic children comes from Hippocrates, although the term “hydrocephalus” was not coined until the writings of Celsus sometime between 25 BC to 50 AD [1, 2]. Though little was initially known about the anatomy and physiology of this captivating problem, anatomists like Galen and Antyllus attempted the earliest surgery with expectedly poor outcomes [1]. Further elucidation of the condition came more recently from the early modern era of seventeenth century anatomists, such as Willis, Monroe, Magendie and Luschka who, among others, began to unravel the architecture of the ventricular spaces and passageways within the brain [1]. The addition of experimentation with material science in the 1800s and early 1900s paired with the Walter Dandy dog model of obstructive hydrocephalus using injection of phenolsulfonphthalein led to our modern understanding of communicating versus non-communicating hydrocephalus [3].

Hydrocephalus is a clinical and neuroradiographic diagnosis characterized by an abnormal accumulation of cerebrospinal fluid which can occur in conjunction with, or in absence of, changes to intracranial pressure. In the latter cases, it is thought that pressure remains normal or low because compensation is occurring elsewhere: either at the expense of the cortical tissue or by the expansion of the skull, or in rare cases, both [4]. The normal flow of CSF is from lateral ventricles through the foramen of Monroe, into the common third ventricle proceeding into the fourth ventricle through the cerebral aqueduct and out of the foramen of Luschka and Magendie into the subarachnoid space. Communicating hydrocephalus occurs when bulk flow is unobstructed and results from a failure to absorb CSF via the normal drainage pathways or an accumulation of CSF due to an over-production [5]. As the name suggests, non-communicating hydrocephalus occurs when there is an obstruction to the normal CSF flow throughout the CNS, and this can be due to a number of precipitating causes. Despite seemingly simple diagnostic criteria such as “communicating” versus “noncommunicating”—among other classifications—the reality is that there remains a burden of clinical heterogeneity among patients with hydrocephalus. Attempts to describe and classify hydrocephalus into discrete categories have often resulted in confusion as there is notable overlap when considering popular categories such as “congenital” or “acquired”. For the purposes of this review, we will consider the classifications of hydrocephalus as either primary (syndromic and/or idiopathic) or secondary to another condition.

Primary hydrocephalus may result from a multitude of genetic factors during fetal development. Genomic discovery-based assays have contributed to our understanding of the causes of primary hydrocephalus as up to 50% genetic. For a review of these causes, please see: [6–11]. Crucially, these genetic mutations have been associated with impaired cellular signaling, development, and proliferation within the central nervous system. Primary hydrocephalus may also result from developmental disorders associated with birth defects in the central nervous system. These may include, but are not limited to, neural tube defects, arachnoid cysts, Dandy Walker syndrome, and Chiari malformation [5]. It is worth noting that there is still an encumbrance of primary hydrocephalus in the clinic which appears to be idiopathic. In the ageing adult population there is an increasing awareness of idiopathic normal pressure hydrocephalus (iNPH), a poorly characterized condition wherein ventriculomegaly causes ageing-related phenotypes such as vision, sleep, cognitive, gait, and continence disturbances progressing to dementia if not treated. While still underdiagnosed, iNPH is cited as occurring in approximately 6% of patients over the age of 80 [12]. The complicated clinical triad of cognitive, gait, and urinary abnormalities contributes to the difficulties in diagnosing iNPH and many neurologists do not consider the role of co-pathology of iNPH with other neurodegenerative diseases. Nonetheless, if started early in symptom progression, treatment by CSF diversion surgery is temporally successful in reversing symptoms in approximately three-quarters of iNPH patients. Though these data are based strictly on Level III trial data and much remains unknown about iNPH, the findings indicate that iNPH is one of the only cases of reversible dementia [13].

Secondary hydrocephalus may result from a multitude of causes including—but not limited to—infection, hemorrhage, and traumatic insult. In developed countries, post-hemorrhagic hydrocephalus (PHH) represents the most common causes of secondary hydrocephalus in pediatric patients. PHH is most often caused by intraventricular hemorrhage (IVH) of prematurity, which is estimated to affect as many as 40% of premature newborns younger than 37 weeks gestation [14]. It is believed that this results from the rupturing of small, delicate vessels along the developing germinal matrix of the brain. These hemorrhages may block or scar the ventricles or clog the drainage pathways along the meningeal vessels [5, 14]. In developing countries, infections of the central nervous system such as meningitis are the more common cause of pediatric hydrocephalus resulting in post-infectious hydrocephalus (PIH) due to inflammation of the ependymal lining and subventricular zone cells, as well as scarring of the CSF drainage sites at the meninges, and an obstruction of CSF drainage or flow [5]. There is also an adult population that is at high risk for developing new, injury-induced post-traumatic hydrocephalus (PTH). Head injury may damage parts of the neurons, glial cells, and blood vessels that can affect CSF production, flow, and/or drainage [5]. Traumatic brain injury (TBI) has increased the number of adults living with post-traumatic hydrocephalus; that number may be as high as two-thirds of current and former Military Service members who suffered moderate-to-severe TBI [15–18].

The burden on the United States healthcare system to treat patients with hydrocephalus is astounding. Pediatric hydrocephalus patients account for hospital charges of $1.4–2.0 billion per year (average), as well as a disproportionate amount of hospital admissions [19]. CSF diversion surgery and shunt revision surgery account for nearly one third of all neurosurgical procedures annually in the United States [20–22]. The average cost of surgical treatment procedures in the United States is well over $35,000 per intervention and the primary payers are private insurers and Medicare. Within the developing world, an increasing diagnosis of pediatric hydrocephalus creates an augmented burden on an underdeveloped health care infrastructure. Recent studies have demonstrated that major barriers to treatment of hydrocephalus in developing nations continue to be poor health outcomes, low patient compliance and follow-up, and a lack of neurosurgically trained healthcare professionals. Importantly, neuroendoscopic procedures have been shown to be the most effective in developing nations because they require less revision and continued care appointments for patients with pediatric hydrocephalus [23].

The majority of the recent review articles on hydrocephalus have focused on specific aspects of pathophysiology, and we direct readers to the following topic reviews: cerebrospinal fluid and ventricular system [24, 25], hemorrhagic hydrocephalus [14, 28, 29], congenital hydrocephalus [10, 14, 26, 27, 29], and idiopathic hydrocephalus [30–32]. The purpose of this review article is to briefly highlight the complexity of hydrocephalus as a condition, review the current treatment paradigms, and discuss associated theories on pathophysiology. Through the discussion on pathophysiology, this review seeks to give context to prior nonsurgical therapies piloted for the treatment of hydrocephalus and provide a critical analysis of targets in preclinical stages. For a historical summary of prior nonsurgical therapies piloted for the treatment of hydrocephalus, please see Del Bigio and Di Curzio [33]. Since the publication of Del Bigio and Di Curzio, over 30 new studies have emerged on targets for the treatment of hydrocephalus. Therefore, this review broadly summarizes the historical evidence on nonsurgical treatments for hydrocephalus and then synthesizes new literature and offers opinion on convergent pathophysiological mechanisms. Finally, this review offers best practices for characterizing drug targets, delineating current targets, and moving the most promising preclinical targets into the next stages. Only by evaluating the convergent pathophysiology of various etiologies of hydrocephalus can we begin to bolster and inform drug development and make an impact on the management of this condition.

## Methods

Articles were gathered utilizing PubMed, Google Scholar, BioRxiv, and MedRxiv search terms: hydrocephalus, drug treatment, inhibition, choroid plexus, ependyma, glymphatics, meninges, blood–brain barrier, cerebrospinal fluid, ventricles, ventricular system, and intracranial pressure. There were no exclusion criteria, thus the searches returned articles of all styles (reviews, primary literature, clinical trials, etc.) and from all publication years. These articles were reviewed via title and abstract to ensure the focus related to hydrocephalus and/or drug therapies to control ICP and ventricle size, and a detailed analysis of these articles is presented via Prisma Algorithm in Supplemental Fig. 1. Articles which were considered to contain content in the target subject were read carefully and relevant citations from the articles were used as supplementary references. These supplementary papers were then subsequently reviewed and read in detail. Further supplementary material was provided by one of the co-authors (J.R.), who has access to clinical data and expert opinion literature. This review article is neither a meta-analysis nor an opinion piece, it fits somewhere in the middle. It is the intent of the authors that this review article provides historical and current background on treatment paradigms for hydrocephalus, discussion of current models for the pathophysiology of the condition, followed by an analysis of the current preclinical research directed to treatments for hydrocephalus.

**Fig. 1:**
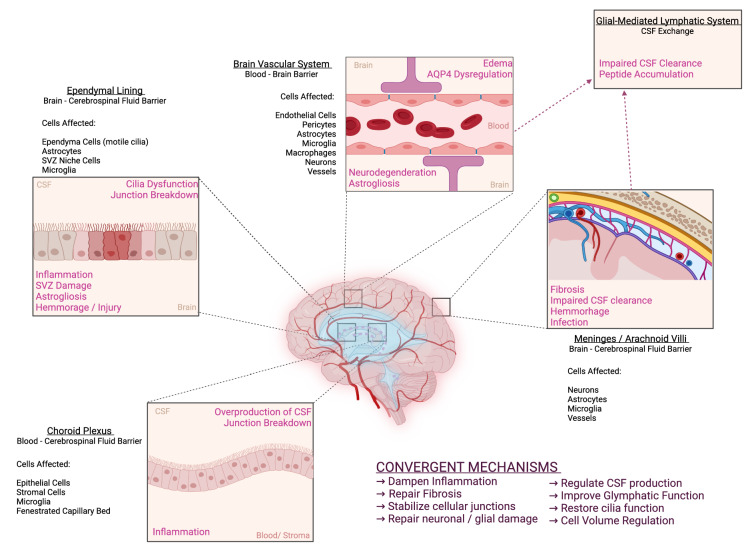
Pathophysiology of hydrocephalus and emerging therapeutic mechanisms. *CSF* cerebrospinal fluid; *SVZ*  subventricular zone; *AQP4*  aquaporin 4. Figure created in BioRender

## Results

### Treatment paradigms

#### Standards of surgical intervention

For patients who are diagnosed with hydrocephalus, there are three tiers of intervention depending on a variety of factors including patient weight, severity of symptoms, and clinical findings. These are described in detail by a systematic review and literature-based guidelines supplement to the *Journal of Neurosurgery* [34]. There are transient nonsurgical interventions, transient surgical interventions, and permanent surgical treatments. Transient, nonsurgical medical interventions include acetazolamide, hyperosmolar therapy with mannitol or hypertonic saline, and hyperventilation. Transient, surgical interventions for hydrocephalus include intermittent and continuous CSF drainage techniques. Intermittent CSF drainage includes serial lumbar punctures, serial transfontanelle aspiration, or placement of a transcutaneous tappable reservoir, while continuous CSF drainage can be achieved by placement of an external ventricular drain (EVD) or creation of a ventriculo-subgaleal shunt (VSGS). The addition of neuroendoscopic lavage (NEL) to remove or dilute the intraventricular debris from infection and hemorrhage is being prospectively studied using the international multicenter “TROPHY” registry [35]. Permanent surgical interventions for hydrocephalus include overcoming an obstruction using neuroendoscopy (e.g., endoscopic third ventriculostomy) or placement of a shunt to effect CSF diversion from production within the ventricle to absorption in a body cavity, most commonly the peritoneum, atrium, or pleural cavity. Permanent CSF diversion is associated with a high risk of failure and often necessitates re-intervention. The gold standard permanent treatment for hydrocephalus is CSF diversion by placement of a shunt.

A shunt has three basic component parts: a ventricular catheter placed in the lateral ventricle, a valve regulating the flow of CSF out of the brain, and a distal catheter that terminates in a cavity. The most common shunt, a ventriculo-peritoneal shunt (VPS), has been well accepted since its inaugural use following the material science advances in the post-WWII era. While the peritoneal cavity is the most common location, ventriculo-atrial (VA) and ventriculo-pleural (VPlS) are acceptable distal targets when the abdomen is unfavorable. In patients with confirmed CSF outflow dysregulation (e.g., iNPH); shunting can be particularly effective at reducing ventricular size and alleviating symptoms [13].

Neuroendoscopy is an alternative to shunting therapy in specifically indicated patients, such as those with obstructive hydrocephalus, and involves placement of an endoscope into the ventricular system for the purposes of addressing primary pathology or changing the bulk flow of CSF. Walter Dandy is credited with the first application of neuroendoscopy to address bulk flow of CSF and popularized endoscopic third ventriculostomy (ETV) in the early 1900s [3]. This procedure overcomes obstructive hydrocephalus at the level of the third ventricle by creation of an ostomy communicating the third ventricle to the subarachnoid space. Initially, ETV morbidity and mortality was very high due to a limited visualization and crude material science with early patient series demonstrating as high as 75% mortality [3].

ETV is undergoing a resurgence in popularity since early 2000s initiated by the work of Dr. Benjamin Warf with patients in sub-Saharan Africa with limited access to shunt hardware [36, 37]. His case series using ETV has shown remarkable control of hydrocephalus of ~ 80% in this population. ETV is sometimes paired with choroid plexus cauterization (CPC) in an attempt to decrease the volume of CSF produced by the choroid plexus of the lateral ventricles. We now understand certain risk factors predisposing patients to fail ETV including young age, non-obstructive etiology, and presence of a shunt. These three factors combine to form the ETV success score (ETVSS) that is used to predict whether it is reasonable to attempt an ETV in selected patients [38].

Despite significant advances in our knowledge of CSF diversion as a management for hydrocephalus, infection and failure of therapy plague the management of these at-risk patients. Notwithstanding multi-billion-dollar-a-year advancements in the medical device industry, new shunts are still wrought with problems. In children, shunts fail with a frequency of up to 50% in the first two years [39–42]. Shunts fail for mechanical reasons (catheter breakage or disconnection), malposition (proximal end not in ventricle, distal end not in abdomen), or obstruction (choroid plexus ingrowth, epithelialization). Each shunt failure requires at least one operation, with additional risk of morbidity and mortality [42]. ETV with or without CPC seems to have a different effect depending on the underlying etiology of the hydrocephalus and long-term failures requiring revision are common. Of the nearly 40,000 surgical interventions for hydrocephalus annually, less than a third are the patient’s first surgery to treat their condition in the United States [19].

Particularly difficult is the counseling of caregivers for young pediatric patients with hydrocephalus towards either shunt or ETV, trying to minimize the procedure related morbidity. The Kaplan–Meier curves for patients with a 60–70% ETVSS cross at 6 months following the index procedure. The index procedure of ETV usually has significant follow-up and imaging which stresses the caregivers and limits their return to normalcy even if it is an effective procedure for hydrocephalus. In contradistinction, VPS placement in the short term does not require that frequent imaging or close follow-up and these caregivers can return to normalcy quickly; however, it is difficult to quantify the lifetime morbidity of frequent imaging and medical concern regarding patients with a VPS. The ethical and behavioral considerations of counseling families for either procedure are outside the scope of this review, but are certainly present during clinical decision-making.

### Historical drug treatments

#### Small molecule interventions

There have been several attempts since the early 1900s to develop nonsurgical interventions, mainly pharmacotherapies, for the treatment of hydrocephalus. These have been elegantly described in detail by Del Bigio and Di Curzio [33], and so this review will provide only a brief summary of their extensive report. The earliest drug intervention recorded in the literature is the use of osmotic diuretics in the treatment of pediatric hydrocephalus. Notably, early studies using theobromine sodium salicylate (Diuretin), a cousin of caffeine, demonstrated robust efficacy in 4 cases of communicating pediatric hydrocephalus and the treatment was relatively well tolerated by the infants [43]. Usually delivered orally in the form of suspensions and powders, Diuretin is a potent vasodilator and diuretic, previously used for the treatment of coronary insufficiency, hypertension, and cardiac and renal edemas. Interestingly, no further clinical studies were performed despite the initially promising results, and neither Diuretin nor its modern homologs are included as a part of the standard medical management of hydrocephalus of any etiology.

Further studies of osmotic diuretics such as isosorbide and glycerol demonstrated similar efficacy in pediatric hydrocephalus. Oral isosorbide, previously indicated for treatment of acute closed angle glaucoma, was found to temporarily control CSF volume and intracranial pressure (ICP) in patients with acquired pediatric hydrocephalus where there was substantial brain mass remaining (cerebral mantle thickness > 20 mm). The treatment was poorly tolerated by most of the patients, resulting in clinically significant dehydration and hypernatremia within 72 h of continuous administration, and so it was not considered an appropriate agent for the medical management of hydrocephalus [44, 45]. Oral glycerol, another osmotic diuretic, was found to promptly reduce intracranial pressure in adults without secondary rebound above baseline and was considered at least transiently effective for patients with high ICP awaiting surgical intervention [46]. The effectiveness of glycerol is comparable to urea and mannitol in reducing cerebral edema and both intracranial and intraocular pressure, though notably without treating hydrocephalus per se [46]. This treatment is utilized today as a transient nonsurgical intervention in patients with hydrocephalus [34, 36]. In the 1970s, a number of studies investigated the utility of various oral cardiac glycosides at sub-toxic doses to reduce CSF production via inhibition of the Na^+^/K^+^-ATPase pump which resides in the luminal membrane of the choroid plexus. The data demonstrated mixed results in the clinic, and at least one group postulated that the arresting of fluid secretion by the choroid plexus is too brief, (complete arrest between 25 and 45 min, with rapid recovery to resting levels at 60 min) rendering it useless as even a transient treatment [47].

Sometime in the latter portion of the 1900s, Diamox and Lasix were adopted as clinical practice in the management of post-hemorrhagic hydrocephalus of prematurity (PHH), as it was thought that the use of a systemic diuretic would decrease brain CSF volume. Diamox, a carbonic anhydrase (CA) inhibitor, was initially proposed because CAII is highly enriched in the choroid plexus and inhibition of CAII reduces serum sodium and bicarbonate, thereby decreasing blood volume and corresponding edema and ICP. Lasix, a loop diuretic, inhibits the sodium–potassium-2-chloride cotransporter 1 (NKCC1) in the choroid plexus, and was postulated to decrease intracellular chloride in the epithelial cells and thus control CSF volume. This treatment was typically administered as a first attempt to control the hydrocephalus or was done in conjunction with the placement of a ventriculo-peritoneal (VP) shunt. However, a systematic review of two clinical trial sites including 193 infants concluded that use of Diamox and Lasix therapy was not safe or effective at treating PHH and therefore should not be recommended for clinical use. The trial data demonstrated that neither Diamox therapy nor Diamox + Lasix combination therapy reduced the risk of death or the need for a VP shunt and furthermore did not improve any neurological, behavioral, or cognitive outcomes at 1 year and conferred additional mortality [48–50]. Additionally, the therapies were found to moderately increase motor impairment and significantly increase the risk of nephrocalcinosis [48–50]. In adults with iNPH, level IV data suggest Diamox treatment modestly improves symptoms and in some cases, prevents the need for shunt insertion, leading to several ongoing clinical trials investigating Diamox as a front line intervention for the treatment of iNPH ([51]; e.g., NCT: 04975269).

Interestingly, a single study of three adults with chronic, poorly managed hydrocephalus saw a complete resolution of all symptoms during a 3- to 12-week treatment with triamterene [52]. Triamterene is a potassium-sparing diuretic which directly inhibits the epithelial sodium channel (ENaC), resulting in control of salt retention in the absence of modulating potassium levels. This study, despite remarkable results, was never followed up and to this day the presence of ENaC in human choroid plexus (and elsewhere in the brain) is widely debated by epithelial cell biologists.

Despite their limited successes, these early pharmacotherapy trials certainly inform researchers about the role of systemic hydration and blood pressure on controlling ICP and CSF volume. Osmotic diuretics do not affect CSF production, but rather, dehydrate the normal brain parenchyma by disturbing osmotic gradients across the normal blood brain barrier. The impact on intracranial hypertension is profound but transient for patients at the end of the spatial exhaustion phase of the compliance curve. Conversely, loop diuretics appear not to treat either intracranial hypertension or CSF volume, despite in theory reducing CSF production. Finally, a single, successful trial of a potassium-sparing diuretic provides evidence for control of sodium levels as a therapeutic target in reducing ICP and associated symptoms. Unfortunately, the confounding variable of systemic drug delivery with effects on multiple organs plagues these studies with poor patient tolerability. The studies of diuretics, while puzzling and difficult to synthesize, demonstrate that control of brain fluid volume and intracranial pressure may be effective in the medical management of hydrocephalus. Nonetheless, the development of more selective compounds with more direct mechanisms of actions remains an open question in the field and is an absolute requirement to show long-term clinical benefit.

#### Anti-inflammatory and anti-fibrotic approaches

In addition to small molecule interventions designed to modulate CSF volume, there have been clinical trials examining pharmaceutical interventions to disrupt inflammation and fibrosis. A large portion of cases of pediatric hydrocephalus occur due to inflammation and fibrosis caused by either hemorrhage or infection. Promising preclinical research suggested that fibrinolytic agents delivered intrathecally, coupled with lavage of remnant blood, could be used to treat PHH. However, in several initial studies [e.g., [53]] as well as a randomized controlled clinical trial titled “DRIFT: Drainage, Irrigation, and Fibrinolytic Therapy” [54], intrathecal fibrinolytic therapy with or without lavage of remnant blood did not improve patient outcomes and slightly increased the risk of secondary hemorrhage.

Anti-inflammatory agents have fared more positively in the treatment of hydrocephalus-associated sequelae. A trial by Shoeman and colleagues showed that dexamethasone improved survivability in young children with tuberculous meningitis, a common cause of post-infectious hydrocephalus, however without changes to ICP or degree of hydrocephalus [55]. A randomized, controlled clinical trial by Thwaites and colleagues confirmed that dexamethasone treatment improved survivability from tuberculous meningitis in adults, presumably by reducing hydrocephalus and preventing infarction, though this was not proven due to study limitations [56]. Treatment with anti-inflammatory steroids has been shown to reduce symptoms associated with hydrocephalus including reduction of headache, nausea, lethargy, seizures, and cognitive deficits, though without preventing the need for surgical intervention [57, 58].

### Pathophysiology

#### Development of the ventricular system

In mammals, the ventricular system first emerges from the enclosure of the neuroectoderm to form the neural tube during neurulation, thus enclosed amniotic fluid forms the precursor to cerebrospinal fluid. This primitive ventricular system is lined with neuroepithelial cells which will eventually proliferate, differentiate, and migrate to form the central nervous system as we understand it. The development of the ventricular system has been recently reviewed [24, 25]. Importantly, the early composition of the CSF and the correct differentiation and migration of neural precursors during this critical period is a possible link to many pathophysiologies observed in primary hydrocephalus. For further reading on the pathophysiology associated with impaired neurogenesis in hydrocephalus, please see: [26, 59–62]. Briefly, primary hydrocephalus has been causally linked via genomic and experimental data to impaired neuro- and glio-genesis during human neonatal development.

#### The blood–CSF interface

**Fig. 2:**
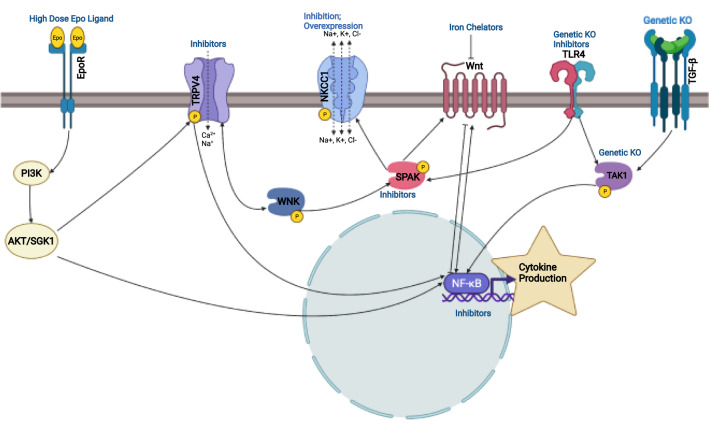
Convergent intracellular signaling in inflammation-related hydrocephalus pathophysiology. *Epo* erythropoietin, *PI3K* phosphoinositide 3-kinase, *AKT* Ak strain transforming kinase / protein kinase B, *SGK1* serum- and glucocorticoid-induced kinase 1, *WNK* with no lysine kinase, *TRPV4* transient receptor potential vanilloid 4, *NKCC1* sodium potassium 2-chloride cotransporter, *NF-kB* nuclear factor kappa-light-chain-enhancer of activated B cells, *SPAK* SPS1-related proline/alanine-rich kinase, *TLR4* toll-like receptor 4, *TAK1* transforming growth factor beta-activated kinase 1, *TGF-β* transforming growth factor beta. Figure created in BioRender

Though there is some dissent within the field, the widely accepted view is that the majority of CSF is produced by the choroid plexus epithelium (CPe), a unique barrier tissue which resides in each of the four brain ventricles. A barrier epithelium surrounds a dense, fenestrated capillary network which is fed from the brain’s arterial blood supply [46]. CSF production and composition is a tightly controlled process which is dependent on specialized channels, transporters, and pumps which alters the plasma filtrate to form CSF [46]. The apical (CSF-facing) surface of the choroid plexus epithelium contains a high density of water-permeable channels as well as electrolyte transporters, which coordinate to secrete CSF into the extracellular milieu [63]. It is thought that CSF production remains independent of hydrostatic pressure, because if Starling’s law of filtration were true, the CSF would appear to be a plasma ultra-filtrate. The composition of the CSF is significantly different than that of plasma, and therefore this indicates that the secretion of CSF is an active and highly regulated process independent of simple osmotic and pressure gradients. Furthermore, recent studies have shown that the composition of CSF is elegantly controlled via development, metabolism, circadian rhythms, and hormones [63–66], indicating the production of CSF an active process. In the context of hydrocephalus, the pathophysiology of the choroid plexus is poorly explored. However, it is known that choroid plexus cell death and corresponding junctional breakdown can occur as a result of oxidative stress and inflammation, leading to dysfunction of CSF production and movement, secretion of signaling molecules, and maintenance of CSF volume [67, 68] as visualized in Figs. 1 and 2.

#### The brain–CSF interface

From the site of production at the choroid plexus, CSF flows throughout the brain ventricular system via a bulk flow model. This process is aided by the presence of motile cilia which beat in a concerted manner to maintain flow of CSF throughout the ventricular system and through the central canal of the spinal cord. The ependymal lining of the ventricular system is a highly specialized, barrier epithelial tissue contiguous with the choroid plexus and of the same neuroepithelial lineage [68]. This epithelial sheet directly contacts the CSF flowing throughout the ventricular system on its apical surface, and directly contacts the brain parenchyma on its basolateral surface. Significantly, the apical surface of these ependymal cells possess bundles of motile cilia which beat in concerted waveforms to propel CSF throughout the ventricular system [68]. It is well characterized that in many forms of hydrocephalus, ependymal cells are denuded or damaged and the lack of motile cilia can precipitate dysfunctional CSF homeostasis as visualized in Fig. 1 [68, 69]. Importantly, restoration of cilia function and repair of damaged ependymal cells has been shown to repair pathophysiological manifestations associated with hydrocephalus in animal models and early human clinical trials [70–72].

#### CSF pulsatility

It has recently been shown that CSF flow throughout the ventricular cavities can be influenced by pulsatile activity of the cerebral vessels, respiration rate, and heart rate [73]. Cardiac-driven pulsations contribute to CSF flow pulsatility across the aqueduct, and this has been confirmed utilizing CINE Flow MRI. However, in the clinic, it appears respiratory rate is, in fact, the major contributor. Dreha-Kulaczewski et al. suggested that the aqueduct is the least favorable portion of the ventricular system to study CSF flow due to its volatility as explained by its exposure to brain movements [74]. In the context of hydrocephalus, another study noted that cardiac-driven pulsatile CSF flow across the cerebral aqueduct was found to be slightly elevated in communicating hydrocephalus, but the authors concluded this was not sufficient to explain the ventriculomegaly [75]. One recent study in the rodent literature confirms that the phenomenon of ventricular wall motion is affected by vasomotion [76]. There is some evidence that pulsatile flow of CSF driven by cardiac forces is unfortunately not recapitulated in *Xenopus* model systems, which are, otherwise, well-established preclinical models of the developing CNS [77, 78].

#### The blood–brain barrier interface

The blood–brain barrier (BBB) is thought to contribute to the extra-choroidal secretion of CSF [79]. The BBB is composed of endothelial cells which line cerebral capillaries, supported by pericytes and surrounded by the endfeet of astroglial cells. These endothelial cells are composed of tight junctions, allowing for the controlled transport of ions, solutes, and cells between the blood cavity and the nervous tissue. Interstitial fluid (ISF), secreted by the BBB, is thought to fill the narrow extracellular space (ECS) between neurons and glia within the parenchyma, complementing the CSF that fills the ventricles and subarachnoid space (SAS). It is thought that CSF and ISF interact in perivascular spaces wherein fluid can flow throughout the brain ECS via convective mechanisms [80, 81]. Increased intracranial pressure and compression of the parenchymal volume due to hydrocephalus has a direct consequence of damage to the BBB endothelial cells and associated support cells [82].

#### CSF drainage pathways

An understanding of conventional CSF drainage pathways was based on the anatomical observation that the human subarachnoid space contains arachnoid villi that project into the venous sinus for drainage. It is currently thought that the arachnoid villi contribute to CSF drainage in conjunction with cervical lymphatic vessels, and the spinal canal [83]. In humans, however, the development of arachnoid villi occurs after birth, and this suggests that during gestation alternative mechanisms of CSF drainage are occurring and these may persist after birth [84].

Unlike other organs, the brain’s lymphatic system is poorly characterized. Recent studies have indicated a role for extracranial lymphatic channels as a mechanism of CSF clearance. The transport of CSF to cervical and spinal lymphatics has been shown to be conserved across several species, which has encouraged further identification of the understudied lymphatic channels of the central nervous system [85].

It is known that the CSF acts as a clearance mechanism for extracellular solutes throughout the CNS, though until recently this mechanism remained undescribed. Major achievements in live imaging and the combination of genetic manipulation and pathophysiology in rodent studies have identified a CSF transport pathway along perivascular tunnels which are surrounded by astroglial cells. This “Glymphatics” system (“Glial-mediated lymphatics”) hypothesis proposes that CSF moves from the SAS into the brain parenchyma along the periarterial space. The CSF then mixes with ISF from the BBB and drains back into the CSF system along perivenous spaces [86]. Studies have indicated that this system depends on the presence of aquaporin water channels and may be responsible for the clearance of peptides such as amyloid and tau from the parenchyma [86, 87]. In the context of hydrocephalus, astrocytic and neuronal damage and death are proposed to impair proper glymphatic drainage and BBB function, at least in part due to dysregulation of aquaporin channels, as visualized in Fig. 1 [87].

#### Intracranial pressure

The CSF volume is an important component for maintaining a stable ICP, and disruptions to rates of CSF secretion and/or drainage can lead to high ICP. In humans, sutural fusion and fontanelle closure at about 18 months of age transforms the pliable calvarium into a rigid, closed intracranial container. The intracranial contents include 10% CSF, 10% arterial and venous blood, and 80% brain tissue. The Monro–Kellie hypothesis relates these volumes to each other, postulating that a change in brain tissue volume, cerebral blood volume, or CSF volume will result in a reciprocal change in one or more of the other components [4]. Homeostatic compensation allows for maintenance of a normal ICP and protects the functionality of the central nervous system. When one of these volumes changes outside of homeostatic regulatory ranges, an ICP elevation occurs. Elevated ICP is common in most forms of secondary hydrocephalus, and reduction of ICP is often used as an indicator of success of treatment intervention. CSF is produced at a rate of about 500 mL/day [63] in adults and is also normally absorbed at the same rate. CSF serves many functions and is the cushioning and nutritive fluid compartment which supports the brain’s complex network of vasculature, neurons, and glia. Increased ICP coupled to a buildup of CSF within the ventricular system can cause secondary injury to the brain parenchyma due to inadequate blood perfusion, oxygenation, and metabolism [64]. Uncontrolled increased ICP in the context of hydrocephalus can result in nausea, vomiting, headaches, vision loss, and, if left untreated, death. These consequences underscore the necessity of maintaining appropriate ICP within the central nervous system. Some types of primary and secondary hydrocephalus notably occur in absence of changes to intracranial pressure. Thus, the discussion of ICP control in the context of hydrocephalus should be prudently revised as an associated symptom requiring treatment, but not a diagnostic hallmark of the condition.

### Targets in preclinical stages

#### Control of brain fluid volume

Preclinical in vitro and in vivo studies have implicated NKCC1 as an important regulator of CSF production leading to the hypothesis that inhibition of NKCC1 would be an attractive target in the treatment of hydrocephalus. However, interpretation of apparently conflicting studies is complicated by lack of agreement in the electrolyte transport field. This is exemplified by the question of whether NKCC1 transports the three electrolytes into the cells, and ultimately toward the blood side of the choroid plexus or whether it is transporting the electrolytes out of the cells, thereby contributing to CSF secretion into the ventricles. The failure of Lasix (furosemide), an inhibitor of NKCC in the treatment of pediatric hydrocephalus argues against the efficacy of systemic inhibition of this cotransporter. Perhaps one of the limitations of the previous clinical studies was route of administration. It is unlikely that oral or intravenous therapies would inhibit NKCC1 at the site of the luminal (CSF-facing) membrane of the CPe. These routes of administration are far more likely to target NKCC1 either peripherally or within the brain vasculature, where the transporter may have alternate functions. Recent theories and studies have evaluated intrathecal delivery of Bumex (bumetanide; a more selective NKCC1 inhibitor) in adult mice to control CSF secretion [88], representing a possible small molecule treatment for hydrocephalus and reinforcing the importance of drug administration and target distribution.

In contrast, another recent article details the neurodevelopmental role of NKCC1 in the choroid plexus. The authors demonstrate that NKCC1 overexpression early in development results in smaller ventricles via the absorption of potassium through the cotransporter and this phenotype persists throughout adulthood in mice [89]. This early NKCC1 overexpression partially attenuates experimental hydrocephalus induced by kaolin injection, suggesting that in the case of pediatric hydrocephalus, drugs that increase NKCC1 activity at the site of the choroid plexus will be advantageous. The authors attribute this inward direction of NKCC1 as a developmental phenomenon due to the absorptive properties of NKCC1 and the choroid plexus during early postnatal development in rodents. Care must be taken in interpreting rodent models of hydrocephalus for drug discovery because these models are limited by differences in gestation and neural development as compared to humans. Postnatal rodent development has been correlated with late gestational stages in humans, and so some of the proposed pharmacotherapies elucidated through perinatal rodent models [89], or conversely, adult rodent models [88], may not translate well to neonates vis-à-vis tolerability, mechanism of action, and functional outcomes.

The NKCC1 studies by Xu et al. [89] and Steffensen et al. [88] form an interesting juxtaposition; one study provides proof-of-mechanism for NKCC1 inhibition in controlling CSF secretion, whereas the other provides proof-of-mechanism for NKCC1 overexpression in controlling CSF secretion. However, the key to understanding these initially contradicting results may be that the choroid plexus is a highly plastic tissue whose role during embryonic and early postnatal development is poorly understood. Much of what we know about the choroid plexus has been elucidated through “adult” models which may not be ideal for studying a primarily infantile condition such as hydrocephalus. In fact, if we consider the early drug trials of Lasix in the treatment of PHH in infants, the studies by Xu et al. [89] may explain why the trials saw net zero or negative effect sizes. If the role of the choroid plexus during early postnatal development is in fact to absorb and regulate potassium levels through the NKCC1 cotransporter (as the authors hypothesize), then the Lasix treatment would have been inhibiting this important mechanism and perhaps accelerating the hydrocephalus phenotype. This finding critically highlights the developmental timeline of late gestation in human development and early postnatal development in rodents as a unique and understudied phenomenon concerning CSF secretion, transporter function, and ion homeostasis. The creation of models which elucidate the role of the choroid plexus as it contributes to CSF secretion throughout development is an absolute requirement for informing future drug discovery for the treatment of hydrocephalus.

Another promising small molecule treatment was the discovery that antagonists of the transient receptor potential vanilloid 4 (TRPV4) channel, a polymodal, non-selective cation channel, reduced progressive ventriculomegaly in a genetic rat model of hydrocephalus [90]. It was proposed that TRPV4 acts as an important signaling hub protein in the luminal membrane of the choroid plexus where it can acutely respond to insult and may be activated in response to ventriculomegaly. The authors hypothesized that since the systemic delivery of the TRPV4 antagonist did not affect ventricle size in wild-type animals, the increased permeability of the various brain barriers due to the hydrocephalus allowed the drug to reach its target in the choroid plexus. TRPV4 is highly enriched in the CSF-facing membrane of the choroid plexus epithelium, but is relatively ubiquitous in the central nervous system, acting to coordinate a variety of intracellular signaling pathways. Interestingly, no adverse effects were seen from the drug treatment despite the ubiquitous nature of TRPV4. Perhaps underscoring this finding are that data that show *Trpv4-/-* null mice and rats do not demonstrate overt phenotypes and are relatively normal, healthy, fertile animals [91–93]. Furthermore, GlaxoSmithKline have reported several clinical trials utilizing TRPV4 antagonists for the treatment of various edema indications, including pulmonary edema, chronic cough, congestive heart failure, and macular edema [[94], NCT02497937, NCT02119260]. In Phase I safety studies, no adverse events have been reported. The promising safety and tolerability data on TRPV4 antagonists in general, suggest that additional preclinical mechanistic studies examining the role for TRPV4 in the pathophysiology of hydrocephalus are crucial.

#### Control of inflammation and fibrosis

Although NKCC1 may be an important regulator of CSF production, an alternative drug target with a stronger preclinical mechanistic basis is the upstream regulatory kinase, SPS1-related proline/alanine-rich serine-threonine kinase (SPAK) that is known to control NKCC1 activity [95]. In a murine model of experimental post-hemorrhagic hydrocephalus, a SPAK inhibitor was found to diminish ventriculomegaly through reducing CSF hypersecretion [96]. Interestingly, the CSF hypersecretion in this murine model of post-hemorrhagic hydrocephalus was found to be due to increased Toll-Like Receptor 4 (TLR4)-mediated inflammation. TLR4 was proposed to regulate SPAK kinase and therefore NKCC1 [95]. Interestingly, both genetic knockout and intrathecal pharmacological knockout of either TLR4 or SPAK was found to attenuate the CSF hypersecretion and therefore treat the hydrocephalus, providing a strong preclinical mechanistic link, at least in mice [95]. Additionally, intrathecal acetazolamide was shown to not affect the hydrocephalus and this preclinical result confirms decades of poor human clinical outcomes. This research represents the convergence of direct control of CSF secretion and inflammation as an effective target in the treatment of hydrocephalus [95].

The SPAK kinase pathway also modulates the Wnt signaling cascade, which is known to be an important modulator of NF-kB-mediated cytokine production [97, 98]. In a rat model of PHH following intraventricular hemorrhage, Wnt signaling was found to be upregulated as a result of increased iron in the cerebrospinal fluid [99]. The PHH was attenuated by treatment with Deferoxamine, an iron chelator, and it was also shown that this treatment improved behavioral outcomes in the PHH rats.

In addition to the heme iron challenge of intraventricular hemorrhage, it has been shown that lysophosphatidic acid (LPA) insult is an important precipitating cause of the development of PHH [100]. In fact, LPA induction alone can create PHH to the same degree as whole blood intraventricular hemorrhage in animal models [100, 101]. Treatment with an LPA_1_ receptor antagonist was effective at preventing PHH following LPA challenge and represents another promising target in the treatment of hydrocephalus.

Given the robust literature suggesting a role for inflammation in the pathophysiology of hydrocephalus, development and repurposing of biologics is an important direction for the treatment of hemorrhagic or infection-related hydrocephalus. In a clinical case of meningitis-induced hydrocephalus, a treatment-resistant infant responded well to a 1-year treatment with Anakinra, a selective IL-1a receptor antagonist. Dampening down of the pro-inflammatory cytokine dramatically resolved the patient’s symptoms and stabilized the progression of the hydrocephalus [102]. This is a promising clinical outcome and represents at least one inflammatory pathway that warrants further exploration in the pathophysiology of hydrocephalus.

#### Neurorepair and neuroprotection

Comparable to Wnt signaling, TGF-β signaling is known to play a critical role in neurodevelopment. Not only do TGF-β overexpressing mice develop severe postnatal hydrocephalus, but there is also a dramatic increase in TGF-β released into the CSF after intraventricular hemorrhage [103, 104]. Due to the ubiquitous nature of the cytokine, TGF-β direct inhibition may not be the most promising target for the treatment of hydrocephalus, but it certainly represents an important group of signaling pathways to be explored for their involvement in the development of hydrocephalus. For example, it was found that intraventricular administration of hepatocyte growth factor (HGF) treats hydrocephalus due to TGF-β overexpression, and this represents a more favorable pharmacological target [105].

Several studies have identified that impaired neurogenesis, gliogenesis, and an upregulation of inflammatory pathways contribute to the development of hydrocephalus [5, 14, 26, 29, 67–69]. Furthermore, it has been shown that damage to the ependymal lining of the ventricles due to blood, inflammation, fibrosis, or trauma, can cause hydrocephalus [67–69]. Recent studies have identified several agents which may protect against this damage and/or restore already damaged tissue. Erythropoietin (Epo), a hormone involved in erythropoiesis, has been shown to be a potent neuroprotective agent with anti-apoptotic, anti-inflammatory, and neurotrophic properties which also helps to decrease free iron and improve neurodevelopmental outcomes [106]. In a kaolin-induced rat model of hydrocephalus, recombinant human Epo was found to reduce the progression of hydrocephalus, decrease microglial activation, and astrogliosis [107]. Additionally, in a rat model of acquired PHH, Epo therapy in combination with melatonin was shown to decrease macrocephaly and ventriculomegaly, as well as prevent matted and missing ependymal cilia, decrease astrogliosis, and increase performance on a neurobehavioral test [71]. Given that Epo is an important regulator of NF-kB signaling, it is also likely to regulate both TLR4-mediated inflammation and SPAK-associated pathways, implicating Epo as not only a neuroprotective agent, but also a potent anti-inflammatory.

#### Advances in stem cell therapies

Recently, the field of stem cell biology and the applications of stem cell transplants have been making promising strides in the precision medicine field. Since inflammation and impaired neurogenesis have been implicated as important causes of the neurodegenerative phenotype of hydrocephalus, stem cell therapy is, theoretically, a viable treatment modality. In rodent studies in 2013 and 2015, it was found that implantation of mesenchymal stem cells (MSCs) in the ventricular wall resulted in the prevention of the progression of hydrocephalus as well as the neurorepair of the injured site [69, 108]. An additional study in 2020 confirmed that MSC implantation in the ventricular wall resulted in a decrease in inflammation, and an increase in neurorepair which rescued the neurodegeneration due to hydrocephalus [109]. Additionally, in 2018 a Phase I clinical trial was completed which evaluated the safety and tolerability of MSC grafts in pediatric hydrocephalus patients. No adverse events were reported, and the therapy was well tolerated by all patients [110].

Though the initial safety studies of MSCs are promising, perhaps more compelling is the grafting of healthy neural stem cells (NSC) and neural progenitor cells (NPC) onto damaged neuroependyma. In a recent study, NSC/NPC explants were cultured into functioning neurospheres and implanted into hydrocephalic mice. These grafts formed functional ependymal motile cilia and developed into neural progenitor cells [72]. Lastly, a recent report piloted the use of explanted choroid plexus as a neuro-restorative therapy. Explanted choroid plexus tissue was found to generate beating cilia in culture, express apical aquaporin-1 and secrete transthyretin, though it did not produce CSF [111]. When implanted into the lateral ventricle of hydrocephalic rats it did not block CSF flow, in fact it maintained beating cilia and secreted important neuromodulatory factors. The authors hypothesized that during choroid plexus cauterization surgery, the choroid plexus could, instead, be removed, cultured, and reimplanted as a ventricular graft. This would allow for a reduction in the production of CSF, as well as maintaining the benefits of neurorepair and neuromodulation that are necessary for improving patient outcomes after surgical intervention [111]. While not strictly non-surgical or non-invasive, stem cell and/or grafting therapies are certainly poised as potent neuro-restorative techniques that may help rescue some of the cognitive and behavioral deficits associated with hydrocephalus and circumvent the repetitive surgeries that seem inevitable in shunt placement.

#### Emerging roles for gene therapy

There has been an increased interest in identifying potential genetic origins of congenital hydrocephalus with the goal of developing gene therapy treatments. Summarized in recent literature, [see 6, 8, 9] there were several genes identified which are causative in primary hydrocephalus in both animal models and in humans. It has been estimated that upwards of 50% of cases of primary hydrocephalus in pediatric patients may have genetic causes [8, 9]. These genes have been identified as important regulators of neuro- and glio-genesis, cell fate, and barrier function, indicating that all these mechanisms are important for the pathophysiology of hydrocephalus. The recent successes of gene therapy for various diseases such as inherited retinal diseases, spinal muscular atrophy, and hemophilia, suggest that new-age gene therapy tools may provide a semi-permanent treatment option for patients with primary hydrocephalus due to genetic mutations [112–114].

#### Convergent mechanisms and the probability of combinatorial treatments

**Fig. 3:**
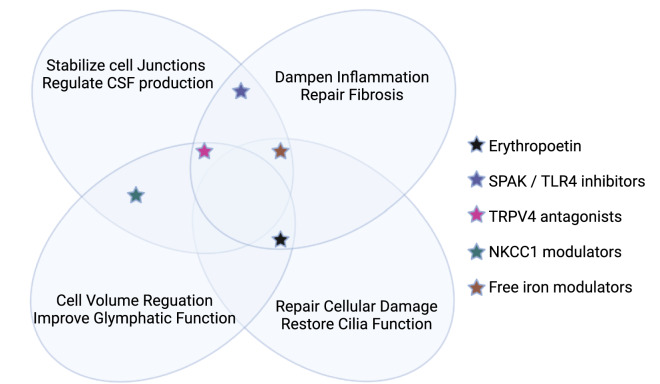
Proposed mechanism of action of preclinical targets. *CSF* cerebrospinal fluid, *SPAK* SPS1-related proline/alanine-rich kinase, *TLR4* toll-like receptor 4, *TRPV4* transient receptor potential vanilloid 4, *NKCC1* sodium potassium 2-chloride cotransporter 1. Figure created in BioRender

**Fig. 4:**
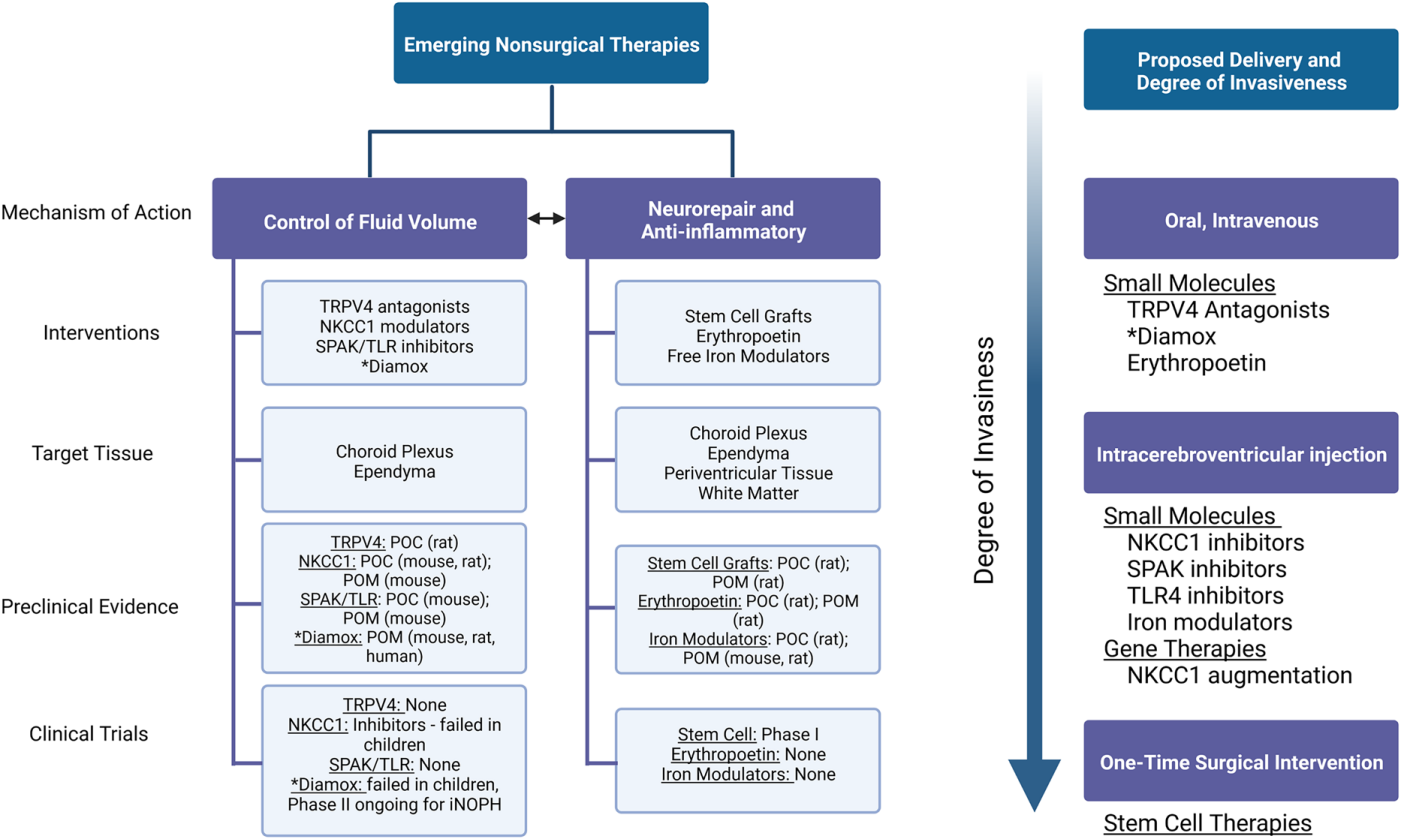
Summary of targets in preclinical stages. *Diamox indicates Diamox treatment for iNPH. *TRPV4* transient receptor potential vanilloid 4, *NKCC1* sodium potassium 2-chloride cotransporter, *SPAK* SPS1-related proline/alanine-rich kinase, *TLR4* toll-like receptor 4, *POC* proof-of-concept, *POM* proof-of-mechanism. Figure created in BioRender

Despite the heterogeneity of the clinical presentation of hydrocephalus, several mechanisms appear to be convergent in the development of the condition (Fig. 1). First, aberrant CSF homeostasis is known to contribute to hydrocephalus through either impaired cilia motility, underabsorption or, less commonly, hypersecretion. Secondly, fibrosis is common in cases of obstructive hydrocephalus and is often found after intraventricular hemorrhage and subarachnoid hemorrhage. Thirdly, impaired neurogenesis and gliogenesis are common hallmarks of pediatric hydrocephalus. Lastly, inflammation is present in most cases of hydrocephalus and is often the precipitating cause of fibrosis and dysregulation of neuronal or glial populations. Therefore, the most effective treatments for hydrocephalus will converge on multiple mechanisms to be effective for the broadest spectrum of patients. Several pharmacological targets have been identified which aim to regulate various aspects of the development of hydrocephalus and thus serve as promising preclinical targets, as visualized in Figs. 3, 4. Epo is not only a neuroprotective agent, but it also prevents fibrosis, reduces free iron, and potentially regulates inflammation. TLR4 inhibitors and SPAK inhibitors not only reduce inflammation, but they also downregulate NKCC1, therefore potentially controlling CSF hypersecretion. TRPV4 inhibition has been linked to a multitude of signaling mechanisms which involve the stabilization of junctional complexes, regulation of transepithelial ion transport, and regulation of cytokine production. It appears combination therapy would be an appropriate treatment for hydrocephalus, controlling many of the molecular mechanisms which can contribute to the development of hydrocephalus. Importantly, a recent preprint article deposited on BioRxiv from Toft-Bertelsen et al. provided some additional insights regarding the role of LPA in hydrocephalus [115]. The authors demonstrated that LPA was significantly increased in the CSF of patients with subarachnoid hemorrhage and also in a rat model of post-hemorrhagic hydrocephalus. The authors then utilized a high-dose LPA treatment (interestingly, 10,000-fold higher than the reference range from the human samples) to induce ventriculomegaly and high ICP associated with PHH. They demonstrated that LPA can act as an agonist of TRPV4 in the choroid plexus and that treatment of their LPA-induced hydrocephalus model with a TRPV4 antagonist ameliorated the ICP and slowed CSF secretion rates. Importantly, through ex vivo studies of choroid plexus, they also showed that this LPA/TRPV4 signaling axis directly modulates NKCC1 activity via SPAK kinase. These data, though preliminary and not yet officially peer-reviewed and published, go a long way to synthesize some of the mechanisms controlling CSF secretion in the choroid plexus.

#### Characteristics of ideal therapy candidates

Before moving into human clinical trials, it is important for research teams to provide a strong preclinical research mechanism and safety profile. Many of the rodent models of hydrocephalus do not accurately reflect the pathophysiology of human hydrocephalus and so in vivo models selected should reflect the human condition as accurately as possible. Drug candidates should be evaluated in both primary hydrocephalus models and secondary hydrocephalus models, as well as communicating and obstructive models of hydrocephalus. Lastly, drug candidates should be evaluated on the same multiple outcomes and surrogate endpoints that are expected of human clinical trials. The use of multiple outcomes and surrogate endpoints is likely to strengthen the rigor of the clinical testing, protect vulnerable patients, and identify whether certain drug candidates will be applicable to all etiologies of hydrocephalus or whether they will only be efficacious in specific types of hydrocephalus.

Drug delivery is also an important aspect of preclinical research (summarized in Fig. 4). Intrathecal delivery circumvents the blood CSF barrier (BCSFB) and is perhaps the most effective way to ensure that drugs reach their target within the choroid plexus or ependymal lining of the ventricles, but it is invasive and carries a certain degree of risk. However, the most convenient, oral formulations confer additional challenges for absorption and distribution and should be thoroughly vetted in preclinical research. Perhaps most translatable is intravenous delivery, as this ensures that the targets will at least get to the blood–brain barrier (BBB) or the blood–csf barrier (BCSFB). However, permeation across the BBB and BCSFB remains a formidable obstacle. Intravenous delivery, though more cumbersome than oral delivery from a patient-provider perspective, is certainly safer and less invasive than intrathecal delivery and there are many cases where regular infusions are standard of care for a given condition. Drug candidates should have adequately characterized pharmacokinetic and pharmacodynamic properties. Ideally, these drug candidates should demonstrate high therapeutic indexes, and this should be conserved phylogenetically. Within the framework of hydrocephalus, ideal drug candidates should also demonstrate convergence on multiple mechanisms of the development of the condition, as this makes it more likely they will succeed in patients of differing etiologies. Finally, ideal drug candidates should be able to be utilized on an as-needed basis and should demonstrate some degree of symptom management or resolution that does not require lifelong dependence.

#### Surgical advancement

Material science has advanced to include common placement of antibiotic impregnated catheters (AICs) which has been shown to independently decrease infection rates [116]. “Smart shunts” are being created which could lead to an in situ understanding of flow or volume rates over time. Multiple industry partners are developing devices which use thermodilution to approximate flow through a distal catheter to aid in the diagnosis of shunt malfunction. Neuroendoscopes have advanced commensurate with optical technology as digital scopes replace older fiberoptic endoscopes. Neurosurgical targets outside of ETV and ETV/CPC continue to remain somewhat limited. Imaging practices continue to use non-ionizing MR and ultrasonography as well as using minimal radiation paradigms. Ultrasonography practices using shear-wave elastography may improve our understanding of intracranial pressure in the future.

## Conclusions

Hydrocephalus is a chronic and lethal condition if left untreated. It affects patients of any age, with thousands of new cases each year in the United States and many more globally. Current treatment of hydrocephalus is almost exclusively surgical, often requiring iterative interventions for the management of the condition. Because of this, the cost-to-treat hydrocephalus is a huge burden on the United States and globally. Thus, the development of pharmaceuticals and nonsurgical interventions to treat hydrocephalus represents a large unmet medical need. There has been limited clinical progress made in this regard in the past 100 years, though there is an encouraging bounty of emerging preclinical data for small molecules, biologics, stem cell therapies, and gene therapies. More complete preclinical proof-of-mechanism and appropriate pharmacokinetic and pharmacodynamic studies in preclinical models will undoubtedly advance the standard of care for the treatment of hydrocephalus and provide a better quality of life for these patients.

## Supplementary Information

**Additional file 1.** PRISMA analysis of article selection for review article

## Data Availability

Not applicable.

## Abbreviations

CSF: Cerebrospinal fluid
PIH: Post-infectious hydrocephalus
PHH: Post-hemorrhagic hydrocephalus
IVH: Intraventricular hemorrhage
TBI: Traumatic brain injury
iNPH: Idiopathic normal pressure hydrocephalus
iIH: Idiopathic intracranial hypertension
VP / VPS: Ventriculoperitoneal (shunt)
VA: Ventriculoatrial
VPIS: Ventriculopleural (shunt)
ETV: Endoscopic third ventriculostomy
ETVSS: ETV success score
CPC: Choroid plexus coagulation/cauterization
ICP: Intracranial pressure
CA: Carbonic anhydrase
NKCC1: Sodium potassium 2-chloride cotransporter 1
ENaC: Epithelial sodium channel
DRIFT: Drainage, irrigation and fibrinolytic therapy trial
CNS: Central nervous system
mL: Milliliter
BBB: Blood–brain barrier
ISF: Interstitial fluid
ECS: Extracellular space
SAS: Subarachnoid space
TRPV4: Transient receptor potential vanilloid 4
SPAK: SPS1-related proline/alanine-rich serine-threonine kinase
AKT: Ak strain transforming kinase/protein kinase B
PI3K: Phosphoinositide-3-kinase
SGK1: Serum-and-glucocorticoid-induced kinase 1
TLR4: Toll-like receptor 4
Wnt: Wingless and Int-1 signaling
LPA: Lipophosphatidic acid
IL-1a: Interleukin 1a
TGF-B: Transforming growth factor beta
HGF: Hepatocyte growth factor
Epo: Erythropoietin
NF-kB: Nuclear factor kappa B
MSC/MSCs: Mesenchymal stem cell(s)
NSC/NPC: Neural stem cells/neural progenitor cells
