# Supplementary figures and images for: Hydrocephalus: historical analysis and considerations for treatment
Source: Eur J Med Res. 2022 Sep 1;27:168. doi: 10.1186/s40001-022-00798-6 (PMC9434947; doi:10.1186/s40001-022-00798-6)

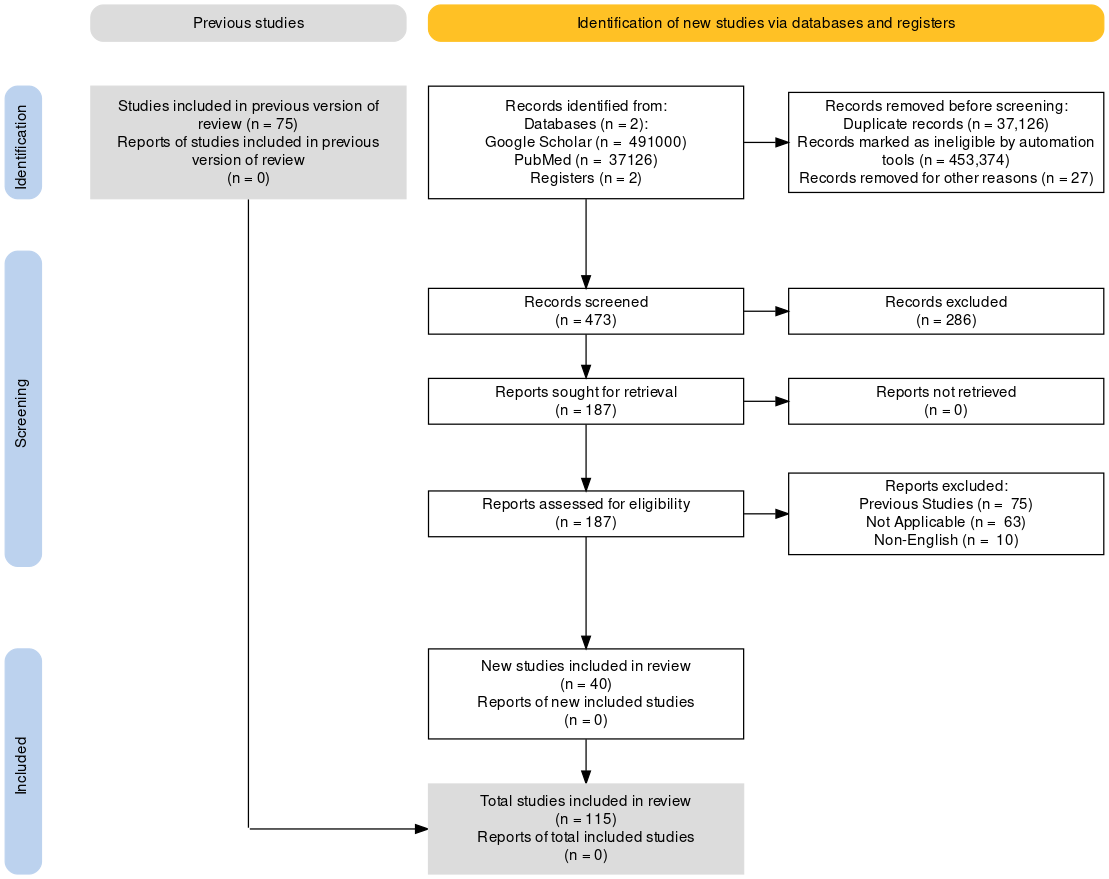

Supplement: Supplementary file 1 — Additional file 1. PRISMA analysis of article selection for review article [file 40001_2022_798_MOESM1_ESM.png]
